# Sensory Processing in Children with Autism Spectrum Disorder and/or Attention Deficit Hyperactivity Disorder in the Home and Classroom Contexts

**DOI:** 10.3389/fpsyg.2017.01772

**Published:** 2017-10-11

**Authors:** Pilar Sanz-Cervera, Gemma Pastor-Cerezuela, Francisco González-Sala, Raúl Tárraga-Mínguez, Maria-Inmaculada Fernández-Andrés

**Affiliations:** ^1^Teaching and Scholastic Organization Department, Faculty of Philosophy and Educational Sciences, University of Valencia, Valencia, Spain; ^2^Basic Psychology Department, Faculty of Psychology, University of Valencia, Valencia, Spain; ^3^Developmental and Educational Psychology Department, Faculty of Psychology, University of Valencia, Valencia, Spain

**Keywords:** Attention Deficit/Hyperactivity Disorder (ADHD), Autism Spectrum Disorder (ASD), higher functions, home and classroom contexts, sensory processing, Sensory Processing Measure (SPM)

## Abstract

Children with neurodevelopmental disorders often show impairments in sensory processing (SP) and higher functions. The main objective of this study was to compare SP, praxis and social participation (SOC) in four groups of children: ASD Group (*n* = 21), ADHD Group (*n* = 21), ASD+ADHD Group (*n* = 21), and Comparison Group (*n* = 27). Participants were the parents and teachers of these children who were 5–8 years old (*M* = 6.32). They completed the *Sensory Processing Measure (SPM)* to evaluate the sensory profile, praxis and SOC of the children in both the home and classroom contexts. In the home context, the most affected was the ASD+ADHD group. The ADHD group obtained higher scores than the ASD group on the Body Awareness (BOD) subscale, indicating a higher level of dysfunction. The ASD group, however, did not obtain higher scores than the ADHD group on any subscale. In the classroom context, the most affected were the two ASD groups: the ASD+ADHD group obtained higher scores than the ADHD group on the Hearing (HEA) and Social Participation (SOC) subscales, and the ASD group obtained higher scores than the ADHD group on the SOC subscale. Regarding sensory modalities, difficulties in proprioception seem to be more characteristic to the ADHD condition. As for higher-level functioning, social difficulties seem to be more characteristic to the ASD condition. Differences between the two contexts were only found in the ASD group, which could be related to contextual hyperselectivity, an inherent autistic feature. Despite possible individual differences, specific intervention programs should be developed to improve the sensory challenges faced by children with different diagnoses.

## Introduction

Recent research has reported that a high percentage of children with different neurodevelopmental disorders such as Autism Spectrum Disorder (ASD) and Attention Deficit/Hyperactivity Disorder (ADHD) show unusual responses to sensory experiences, compared to the responses offered by typically developing children with the same chronological age (Cheung and Siu, 2009; Wiggins et al., 2009; Watts et al., 2016; Little et al., 2017). According to Sensory Integration Theory (Ayres, 1979), these unusual responses are due to some type of dysfunctionality (or difference) involving the registration of sensory information, its modulation, its discrimination, the internal organization and/or the integration of sensory input. Sensory processing (SP) refers to the way the central and peripheral nervous systems manage incoming information from the different sensory modalities, which include the internal modalities of proprioception and vestibular system, and the classical external senses of vision, hearing, taste, smell, and touch (encompassing the latter the broader term of somatosensory senses).

Three types of SP disorders are distinguished: (1) Sensory modulation disorders, which affect the regulation of the level or intensity of the response that occurs in the presence of the sensory information, thus differentiating between over-responsiveness, under-responsiveness and sensory seeking, (2) Sensory discrimination disorders, which affect the ability to distinguish and identify sensory inputs, and (3) Sensorimotor integration disorders, which involve a difficulty in transforming sensations into motor responses, including postural disorders with a sensory basis and developmental dyspraxia, in which ideation and motor planning are compromised, producing difficulties in learning new motor tasks.

Thus, because sensory information forms the building blocks for higher-order cognitive functions (Baum et al., 2015), a neurological dysfunction at the level of SP could contribute to impairments in higher functions, such as praxis. Praxis is the ability to conceptualize or ideate, plan and organize movements in order to carry out unfamiliar motor tasks, and it has two aspects: ideation (the ability to create a conceptual or mental image of a novel task) and motor planning (the ability to organize and plan novel actions) (Parham et al., 2007). Therefore, difficulties with praxis –as happens in dyspraxia- are related to poor performance on activities that require motor skills and flexible problem solving.

Likewise, sensory difficulties and sensorimotor integration difficulties, such as dyspraxia, could also contribute to impairments in higher-order social functions (Baum et al., 2015). For example, poor motor planning skills can limit the ability to expand play repertoires or engage with others (Parham et al., 2007). Thus, in the case of children, many of the physical games they often play in the school playground require sensorimotor integration skills that imply the need to continually devise and plan new motor responses, so that if the child presents praxis difficulties, he or she will find it difficult to integrate into the others game's and this will complicate the child's social participation.

Impairment in sensorimotor skills can keep children from executing successful adaptive responses to situational demands and engaging meaningfully in daily activities (Jasmin et al., 2009). Moreover, research and first-person accounts have revealed that impairments in SP may be related not only to children's SOC difficulties (Miller Kuhaneck and Britner, 2013; Roley et al., 2015; Chien et al., 2016), but also to difficult temperamental characteristics (Brock et al., 2012), sleep problems, and behavioral and emotional problems (Reynolds et al., 2012). These problems can affect not only the children's personal functioning, but also their families' daily routines, leading to higher levels of parental stress than what is found in parents of children without sensory challenges (Ben-Sasson et al., 2013). Hence, it seems important to study more closely the sensory challenges that these children experience in order to improve their personal and family quality of life.

Much of the research on SP has focused mainly on the study of the ASD population, as the current literature suggests that SP impairments are highly prevalent in children with this disorder (Leekam et al., 2007). In addition, the inclusion of sensory difficulties in the DSM-5 criteria (American Psychiatric Association, 2013) has led to an increasing interest in this emerging research area. Literature reports that these difficulties affect the entire spectrum, although a positive relationship has been shown between sensory dysfunction and the severity of ASD in children, so that the greater the sensory dysfunction, the greater the severity of the autism symptomatology (Ashburner et al., 2008; Sanz-Cervera et al., 2015). Sensory impairments in autism are present from toddlers to adults (McCormick et al., 2016), and they are significantly related to stereotyped interests and behaviors (Wiggins et al., 2009).

Among the different sensory modalities, the most affected ones are usually hearing and touch (Tomchek and Dunn, 2007; Ashburner et al., 2008; Wiggins et al., 2009; Fernández-Andrés et al., 2015), especially auditory filtering and tactile sensitivity, which has also been found using objective direct assessment (Tavassoli et al., 2016), performance-based measures (Stewart et al., 2016), and assessment that combines clinical-administered observation and caregiver interviews (Siper et al., 2017). This impairment has been found to influence the severity of the restricted and repetitive behaviors displayed by people with ASD (Kargas et al., 2015).

Regarding higher functions, children with ASD usually present difficulties with praxis (Roley et al., 2015). Thus, motor skills requiring adjustments in initiation, timing, sequencing, speed, and direction of movement are usually difficult for them. This poor performance on activities that require motor skills and flexible problem solving is probably associated with one of the diagnostic criteria for ASD (according to DSM-5), that is “the presence of restricted, repetitive patterns of behavior, interests, or activities” (American Psychiatric Association, 2013).

Last, children with ASD commonly have greater cognitive functioning limitations at high levels of information processing, including social skills (Jasmin et al., 2009; Miller Kuhaneck and Britner, 2013; Miguel et al., 2017), as in the case of games and interactions with other people. The social impairments are expected because one of the diagnostic criteria for ASD (according to DSM-5) is the presence of “persistent deficits in social communication and social interaction across multiple contexts” (American Psychiatric Association, 2013).

Regarding the ADHD population, previous research has found that the SP and modulation patterns of children with this disorder are significantly different from those of typically developing children, using not only behavioral measures (Cheung and Siu, 2009; Engel-Yeger and Ziv-On, 2011; Pfeiffer et al., 2015), but also physiological assessments (Mangeot et al., 2001; Parush et al., 2007). These differences are related to some symptoms of the disorder (Cheung and Siu, 2009), such as inattention, distractibility, hyperactivity, impulsivity, poor adaptability, and so on.

The sensory modalities that appear to be most affected in children with ADHD are vestibular -which has been associated with attentional difficulties (Shum and Pang, 2009)-, proprioceptive (Jung et al., 2014), and tactile processing (Parush et al., 2007; Ghanizadeh, 2008). Some authors have suggested that vestibular and proprioceptive problems in children with ADHD may be related to difficulties in visual processing (Shum and Pang, 2009; Jung et al., 2014). SP impairments have been associated with behavioral problems presented by children with ADHD in different contexts (Dunn and Bennett, 2002). These problems can include anxiety (Reynolds and Lane, 2009), academic achievement problems (Davis et al., 2009), disruptive behavior disorders, and even aggression and delinquency (Mangeot et al., 2001).

Regarding higher functions, children with ADHD usually present sensorimotor and praxis difficulties (Davis et al., 2009; Pfeiffer et al., 2015). Although difficulties in praxis have been found to be associated with the hyperactivity and impulsivity symptomatology (Pfeiffer et al., 2015), it is difficult to determine whether praxis impairments are related to underlying SP dysfunction or to executive dysfunction, a hallmark of ADHD.

Last, regarding SOC, the highest level of functioning, children with ADHD also usually present difficulties in their relationships with others, probably secondary to their impulsive behavior. In fact, their SOC difficulties also have been found to be associated with the hyperactivity and impulsivity symptomatology (Pfeiffer et al., 2015). In addition, social difficulties are well-documented among children with ADHD, being considered a social disability by some researchers (Gentschel and McLaughlin, 2000).

Some studies have found specific patterns of SP consistent with the diagnostic criteria for ASD and ADHD (Cheung and Siu, 2009; Clince et al., 2016). Nonetheless, the high comorbidity rate between ASD and ADHD (Kern et al., 2015) makes it difficult to establish specific patterns of SP for each disorder. In fact, these neurodevelopmental disorders (ASD and ADHD) share some patterns of SP impairments, such as deficits in somatosensory processing, which are manifested as tactile defensiveness (Parush et al., 2007; Tomchek and Dunn, 2007). Regarding higher functions, they share difficulties in motor abilities (Biscaldi et al., 2015), communication, and social skills (Cascio, 2010).

With the possibility of a comorbid ASD and ADHD diagnosis, recognized by the DSM-5 (American Psychiatric Association, 2013), new studies with this comorbid population are needed because very little research has been conducted to date about sensory issues and higher functions such as praxis and SOC. Emerging studies suggest that children with a comorbid ASD+ADHD diagnosis have poorer SP, motor skills, and adaptive behaviors than those with ADHD alone (Mattard-Labrecque et al., 2013). However, it has been found more planning problems in ASD than in ADHD and ASD+ADHD (Unterrainer et al., 2016). Regarding SOC, it has been found more social functioning difficulties in children with ASD+ADHD than in those with ASD alone (Rao and Landa, 2014).

The main objective of the present study was to compare the characteristics of SP, praxis, and SOC of four groups: a group of children with ASD, a group of children with ADHD, a group of children with a comorbid ASD+ADHD diagnosis, and a group of children with typical development. To our knowledge, no published studies have compared SP and other high functions among these four groups. This comparison may make it possible to elucidate different sensory patterns in each disorder, which could help to tailor the interventions, depending on the difficulties the children present in each disorder. It is also important to study the SP, praxis, and SOC of these children in different contexts, as each context contains unique characteristics that can support children and/or create challenges to their performance (Dunn et al., 2002). In addition, the literature on multiple informants indicates that when parents and teachers are asked the same question, the correlations between the answers are low (De los Reyes and Kazdin, 2005). Research to date has only analyzed the sensory difficulties of children with ASD in the most important primary socialization contexts, the family and the school (Parham et al., 2007; Brown and Dunn, 2010; Lai et al., 2011; Fernández-Andrés et al., 2015), with teachers reporting greater dysfunction than parents (Fernández-Andrés et al., 2015). Apart from the comparison of SP in children with ASD, we have not found any other studies conducted in children with ADHD and/or an ASD+ADHD comorbid diagnosis that compared their SP characteristics in different contexts.

These research gaps suggest the need for more specific investigation. Therefore, the aims of this study were: (1) to compare the characteristics of SP, praxis, and SOC of the four groups in the home context (information reported by parents); (2) to compare the same characteristics of the four groups in the classroom (information reported by teachers); and, (3) to compare –in each group separately- the characteristics reported by parents to those reported by teachers.

Based on results from previous studies, we hypothesize that the three groups of children with neurodevelopmental disorders will obtain higher levels of dysfunction than the Comparison Group (CG) in both contexts. Additionally, we expect the ASD+ADHD Group to be the most affected, so that the profiles of this comorbid group and the CG are expected to be the most different.

As for specific sensory patterns in each disorder, it is expected that the most affected sensory modalities in children with ASD would be hearing and touch, whereas in children with ADHD it is expected that the most affected modalities would be body awareness (BOD) and balance and motion, as well as vision and touch. Regarding the higher functions in each disorder, it is also expected that praxis would be equally affected in both disorders, but SOC would be more affected in children with ASD because the presence of social difficulties is a hallmark of ASD.

As for the comparison among the three groups with neurodevelopmental disorders, we expect an additive effect in the comorbid group that would lead to obtain higher levels of dysfunction than in the other two groups with neurodevelopmental disorders.

When comparing contexts, we hypothesize that the three groups of children with neurodevelopmental disorders will obtain higher levels of dysfunction in the classroom context than in the home context, considering the greater demands of school assignments, and teachers' opportunities to compare children's functioning with that of their peers, as well as certain environmental factors characteristic of the classroom context, such as stimulation overload produced by excessive noise, and unpredictable physical contact when working cooperatively (Ashburner et al., 2008).

## Materials and methods

### Participants

The participants in this study were the parents and teachers of a total of 90 children between 5 and 8 years old, who were divided into four groups: The ASD Group (*n* = 21), the ADHD Group (*n* = 21), the ASD+ADHD Group (*n* = 21), and the CG (*n* = 27).

#### ASD group

The ASD Group was composed of 17 males and 4 females who had a clinical diagnosis of ASD. They were diagnosed by neuropediatric services from different hospitals in the national health system, according to the criteria of the DSM-IV-TR (American Psychiatric Association, 2000), and they met the diagnostic criteria for level 2 of the DSM-5 (American Psychiatric Association, 2013). These neuropediatric services were responsible for checking compliance with these diagnostic criteria. They referred the children who met the diagnostic criteria to early care units, where the diagnosis was confirmed using a more specific instrument, the Autism Diagnostic Observation Schedule (ADOS; Lord et al., 2000), which was applied by specialized psychologists who had the official accreditation to use this instrument. Moreover, all of them obtained an Autism Index (AI) score ≥85 on the Gilliam Autism Rating Scale, Second Edition (GARS-2), indicating a high likelihood of the disorder (Gilliam, 2006). Children included in the ASD group did not meet the diagnostic criteria for ADHD.

#### ADHD group

The ADHD Group was composed of 18 males and 3 females who had been clinically diagnosed with a combined ADHD presentation by neuropediatric services, according to the criteria of the DSM-IV-TR (American Psychiatric Association, 2000). All of them showed the presence of six or more inattention symptoms and also six or more hyperactivity/impulsivity symptoms, based on information provided by both parents and teachers; persistence of symptoms for more than 6 months; and the appearance of symptoms before the age of 7. Children included in the ADHD group did not meet the diagnostic criteria for ASD.

#### ASD+ADHD group

The ASD+ADHD Group was composed of 20 males and 1 female who met the same inclusion criteria as both the ASD and ADHD Groups.

#### Comparison group

The CG was composed of 19 males and 8 females who had not received any type of clinical diagnosis.

All of the children attended the same schools. Children from the ASD Group and the ASD+ADHD Group were attending schools with specific classrooms where the Treatment and Education of Autistic and Related Communication Handicapped Children (TEACCH) methodology was used. These are special classrooms integrated in regular state schools in Valencia (Spain), where students with disorders affecting language and communication are enrolled. In these classrooms there are a maximum of 8 children attended by three specialists: a special education teacher, a hearing and language teacher and an educator. These children are not all the time in these special classrooms, but they share their timetable both in these classrooms and in their corresponding mainstream classroom, where they are usually accompanied by one of the specialists who work in the special classrooms. Children from the ADHD Group and the CG, however, were attending the same schools as the children in the ASD and ASD+ADHD Groups, but in the regular modality.

Table 1 includes the children and family's demographic information in each group. The mean age of all the children was 6.32 years (*SD* = 1.11), and the mean non-verbal IQ measured by Raven's Colored Progressive Matrices Test (Raven, 1996) was 98.72 (*SD* = 16.84). No statistically significant differences were found among the four groups of children on gender (χ^2^ = 5.23; *p* = 0.156*;* η^2^ = 0.239), non-verbal IQ [*F*_(3, 86)_ = 0.75; *p* = 0.523; η^2^_*p*_ = 0.026], or chronological age [*F*_(3, 86)_ = 2.03; *p* = 0.116; η^2^_*p*_ = 0.066]. Table 1 also includes the mean number of Inattention and Hyperactivity/Impulsivity symptoms reported by the children's parents and teachers, who answered the items on the behavioral rating scale from the DSM-IV-TR (American Psychiatric Association, 2000).

**Table 1:** Children and parents' demographic information.

	**ASD group (*n* = 21)**	**ADHD group (*n* = 21)**	**ASD+ADHD group (*n* = 21)**	**Comparison group (*n* = 27)**
**CHILDREN'S GENDER**				
Male	17 (81%)	18 (85.7%)	20 (95.2%)	19 (70.4%)
Female	4 (19%)	3 (14.3%)	1 (4.8%)	8 (29.6%)
Mean age (*SD*)	6.06 (1.09)	6.81 (1.10)	6.15 (1.04)	6.28 (1.11)
Mean non-verbal IQ (*SD*)	103.43 (17.39)	97.43 (14.38)	96.19 (18.34)	98.04 (17.21)
Mean inattention^a^ *(SD)*	4.38 (2.67)	6.76 (1.87)	8.29 (0.85)	93 (1.33)
Mean hyperactivity/Impulsivity^a^ *(SD)*	3.57 (1.99)	6.10 (1.87)	7.62 (1.02)	1.59 (1.67)
**PARENTS' RESPONSE**				
Father	4 (19%)	4 (19%)	1 (4.8%)	6 (22.2%)
Mother	17 (81%)	17 (81%)	20 (95.2%)	21 (77.8%)
Mean parental age *(SD)*	40.10 (4.35)	39.48 (4.57)	37.95 (3.96)	39.37 (5.02)
**PARENTS' EDUCATION LEVEL**				
Elementary education	5 (23.8%)	5 (23.8%)	8 (38.1%)	8 (29.6%)
Intermediate education	11 (52.4%)	11 (52.4%)	5 (23.8%)	8 (29.6%)
Higher education	5 (23.8%)	5 (23.8%)	8 (38.1%)	11 (40.7%)
Mean number of children (*SD*)	1.57 (0.60)	1.81 (0.40)	1.95 (0.67)	1.96 (0.59)

a *Mean number of inattention and hyperactivity/impulsivity symptoms reported by parents and teachers (DSM-IV-TR)*.

Regarding families, around 80% of the participants in each group were mothers. The mean age of the parents was 39.23 (*SD* = 4.52; range: 25–50). No statistically significant differences were found among the four groups of parents on gender (χ^2^ = 2.91; *p* = 0.405; η^2^ = 0.156) or age [*F*_(3, 86)_ = 0.84; *p* = 0.474; η^2^_*p*_ = 0.029]. The educational level of the parents was similar in the four groups, and the mean number of children in the family was about 1.80.

A total of 35 teachers participated in the study, of whom 18 were Therapeutic Education or Hearing and Language Teachers in the TEACCH classrooms who completed the questionnaires about the children in the ASD and ASD+ADHD Groups, and 17 were the mainstream classroom teachers who completed the questionnaires about the children in the Comparison and ADHD Groups. Most of the teachers were females, with the exception of two males, but no statistically significant gender differences were found between the two groups of teachers (χ^2^ = 0.002; *p* = 0.967). The age range of the teachers was from 26 to 60, with statistically significant differences in age between the two groups [*F*_(1, 33)_ = 11.39; *p* = 0.002; η^2^_*p*_ = 0.257], as the teachers of the ASD and ASD+ADHD Groups were younger than the teachers of the ADHD and CGs. Regarding educational level, the teachers in the ASD and ASD+ADHD Groups had more academic training than the teachers in the other groups, and this difference was statistically significant (χ^2^ = 6.278; *p* = 0.043; η^2^ = 0.424). All the teachers had between 5 and 36 academic years of teaching experience, with teachers in the ADHD and CGs having more experience than the teachers in the other groups [*F*_(1, 33)_ = 8.48; *p* = 0.006; η^2^_*p*_ = 0.204]. Teachers in the ASD and ASD+ADHD Groups had also more academic years of contact with their students than teachers in the ADHD and CGs [*F*_(1, 33)_ = 8.86; *p* = 0.005; η^2^_*p*_ = 0.212].

### Ethics statement

This study is part of a broader investigation that was approved and funded by the University of Valencia, and it had the official and written authorization of the Valencian Government. All of the Valencian state schools with TEACCH integrated classrooms were invited, via an informative meeting, to participate in the research. From the schools that voluntarily agreed to participate, some classrooms of 5–8-year-old children were selected. The parents of the selected children gave written informed consent to participate in the research.

### Procedures

Each child's non-verbal IQ was individually evaluated by the school psychologist in a noise and distraction-free office. Parents and teachers were asked to participate in an interview with the school psychologist in order to provide demographic information, and they filled out the *Behavioral Rating Scale of Inattention and Hyperactivity/Impulsivity* from the DSM-IV-TR (American Psychiatric Association, 2000), as well as the *Sensory Processing Measure (SPM)* questionnaires. Parents from the ASD and ASD+ADHD Groups also provided information about autism severity by answering the questions on the GARS-2 (Gilliam, 2006).

### Measures

#### Raven's colored progressive matrices (CPM), Raven, 1996

This is a non-verbal scale that measures the test-taker's reasoning ability, providing an estimation of the deductive capacity and the “g” factor of general intelligence. It contains 36 elements, and the child must choose missing pieces from a series of 6–8 elements. The scale is administered to children between 4 and 9 years old. We used the non-verbal IQ score provided by the test.

#### Gilliam autism rating scale, second edition (GARS-2), Gilliam, 2006

This screening scale provides a norm-referenced measure that helps to identify autism and estimate its severity. It can be filled out by professionals or parents of people between 3 and 22 years old. The scale consists of 42 items, responded to on a Likert-type scale, which measure the three characteristic domains adopted by the DSM-IV-TR diagnostic criteria (American Psychiatric Association, 2000): Stereotyped Behavior, Communication, and Social Interaction. The combined scores on these subscales yield an AI score (*M* = 100 and *SD* = 15), with higher scores indicating a greater degree of autism, so that three categories are established: *Improbable Autism* (AI score below 70), *Possible Autism* (AI score from 70 to 84), or *Probable Autism* (AI score equal to or >85). The GARS-2 is a widely-used tool to assess ASD symptoms, and it has been adapted and validated in different countries, with results showing good psychometric characteristics. For the Spanish version, the scale's internal consistency was high (Cronbach's alpha = 0.94 for the AI), and the scale's criterion validity with the Autism Behavior Checklist was also high (0.94).

#### The *Sensory Processing Measure (SPM)*, Parham et al., 2007

This is an integrated system of rating scales for the assessment of SP issues, praxis, and SOC in elementary school-aged children (ages 5–12). Each item is rated in terms of the frequency of the behavior on a 4-point Likert-type scale. The original SPM consists of three forms that evaluate the child's functioning in different contexts. In this study, we specifically used a Spanish translation of the original *SPM-Home Form* and *SPM-Main Classroom Form*. Both forms yield several norm-referenced standard scores corresponding to the different scales of the instrument: Social Participation (SOC), Planning and Ideas (PLA), Vision (VIS), Hearing (HEA), Touch (TOU), BOD, and Balance and Motion (BAL). The last two subscales refer to internal sensory modalities (proprioception and vestibular system, respectively). From the scores obtained on the five sensory system subscales –and additional items representing taste and smell processing- a total score called Total Sensory Systems (TOT) can be obtained. Despite the terminology used on the measures referring to the sensory modalities (vision, hearing, touch…), it must be kept in mind that the subscales assess the impairments (or differences) referred to the SP. Thus, it is not the sensory pathway, but rather the way in which the information related to a particular sensory modality is processed. On the other hand, the SOC and PLA subscales represent higher functions, where SOC (the ability to engage with others) is the subscale that measures the highest function, and PLA is the praxis subscale, which includes items about motor planning (e.g., “Fails to complete tasks with multiple steps”), and items about ideation (e.g., “Unable to solve problems effectively”).

The assessment of the sensory modulation vulnerabilities -such as over-responsiveness, under-responsiveness and sensory seeking- is not included in the norm-referenced standard scores corresponding to the scales of the SPM, although an item-by-item analysis would allow it. Last, the standard score for each subscale makes it possible to classify the child's functioning into one of three interpretive ranges: *Typical* (*T*-score range 40–59); *Some Problems* (*T*-score range 60–69); and *Definite Dysfunction* (*T*-score range: 70–80). Both forms share many structural and interpretative similarities, and so it is possible to compare different contexts. Both questionnaires present high internal consistency (Cronbach's alphas range from 0.75 to 0.95). Regarding validity, the different SPM subscales present correlation indexes from 0.2 to 0.5 with the subscales of the Sensory Profile and the Short Sensory Profile (Dunn, 1999).

#### Behavioral rating scale of ADHD symptomatology from the DSM-IV-TR American Psychiatric Association, 2000

This questionnaire asks parents and teachers about the presence of ADHD symptoms in the child, using the diagnostic criteria included in the DSM-IV-TR (American Psychiatric Association, 2000). It is composed of 18 items, of which 9 refer to the presentation of symptoms associated with inattention, and the other 9 refer to the presentation of symptoms associated with hyperactivity/impulsivity. For each child, we consider the number of inattention and hyperactivity/impulsivity symptoms reported by both the parents and the teacher.

#### Questionnaires developed by the authors

We developed two different questionnaires to ask parents and teachers about some socio-demographic questions (see Table 1).

#### Data analysis

Analyses were performed with the SPSS statistical package, version 23 for Windows. First, the distributions of continuous dependent variables were examined for normality with the Shapiro-Wilk test. Second, two multivariate analyses of variance (MANOVA) were carried out to compare the characteristics of SP, praxis, and SOC of the four groups: one MANOVA to compare the four groups in the home context (parent report) and another MANOVA to compare the four groups in the classroom context (teacher report). Additionally, because the scores obtained on the TOT subscale are the sum of the scores obtained on the different sensory subscales, two ANOVAS were performed to compare the four groups on the TOT subscale: one ANOVA for the home context and another ANOVA for the classroom context. In order to control the probability of type I error, we introduced a correction factor of critical *p* values when performing multiple comparisons, using a step-down method: the Holm-Bonferroni sequential correction (Holm, 1979). Third, to compare the parent report with what the teachers reported in each group, (MANOVA) for repeated measures were performed.

## Results

### Group differences in the home context

Statistically significant differences among the four groups were found as revealed by both the MANOVA performed with the scores on the *SPM-Home Form* [Wilk's Lambda (λ) = 0.304; *F*_(7, 21)_ = 5.64; *p* < 0.001; η^2^_*p*_ = 0.328], and the ANOVA performed with the scores on the TOT subscale [*F*_(3, 86)_ = 24.13; *p* < 0.001; η^2^_*p*_ = 0.457].

As Table 2 shows, the parents of the children with a neurodevelopmental disorder (ASD and/or ADHD) evaluated their children's characteristics of SP, SOC, and praxis as significantly more dysfunctional than the parents of the children in the CG, except on the TOU, BOD, and BAL subscales, where there were no statistically significant differences between the CG and the ASD Group, and the HEA subscale, where there were no differences between the CG and the ADHD Group.

**Table 2:** T-score means, standard deviations, and *F*-values for SPM–home form subscales.

		**ASD group**	**ADHD group**	**ASD+ADHD group**	**Comparison group**	***F*_(3, 86)_**	**η^2^*_*p*_***	**Group differences**
VIS	*M*	59.29	60.19	66.38	50.04	15.11^**^	0.345	C<ASD, ADHD, ASD+ADHD
	*SD*	8.52	9.83	6.41	7.26			
HEA	*M*	62.00	56.52	64.95	48.96	16.11^**^	0.360	C<ASD, ASD+ADHD
	*SD*	9.37	9.66	7.14	8.15			
TOU	*M*	58.00	63.81	61.86	50.81	8.57^**^	0.230	C<ASD+ADHD, ADHD
	*SD*	8.88	13.64	9.10	6.57			
BOD	*M*	56.81	65.71	64.29	51.15	19.69^**^	0.407	C<ASD+ADHD, ADHD;
	*SD*	5.79	10.47	5.75	7.01			ASD<ASD+ADHD, ADHD
BAL	*M*	55.29	61.62	62.00	48.48	9.93^**^	0.257	C<ADHD, ASD+ADHD
	*SD*	9.77	14.36	7.97	6.81			
TOT	*M*	59.86	66.62	65.81	50.11	24.13^**^	0.457	C<ASD, ASD+ADHD, ADHD
	*SD*	7.77	10.12	6.76	5.89			
SOC	*M*	61.86	58.61	69.24	50.04	21.94^**^	0.433	C<ADHD, ASD, ASD+ADHD;
	*SD*	11.42	7.02	6.96	7.26			ADHD < ASD+ADHD
PLA	*M*	59.19	61.91	65.86	48.11	17.68^**^	0.381	C<ASD, ADHD, ASD+ADHD
	*SD*	10.28	10.44	7.67	7.55			

VIS, vision; HEA, hearing; tOU, touch; BOD, body awareness; BAL, balance and motion; TOT, total sensory systems; SOC, social participation; PLA, planning and ideas. A+A = ASD + ADHD Group; C, comparison group.

** *p < 0.013 Holm-Bonferroni correction of critical p-values when performing multiple comparisons*.

In order to obtain sensory profiles of each group, we analyzed the percentages of scores obtained by the four groups in each of the three SPM interpretative ranges. In this context, we noted that 93% of the CG obtained scores within the *Typical* range, whereas a small percentage (about 7%) obtained scores within the *Some Problems* and *Definite Dysfunction* ranges, indicating some difficulty. By contrast, the ASD+ADHD Group obtained the highest scores (about 74%) within the *Some Problems* and *Definite Dysfunction* ranges, whereas small percentages were obtained in the *Typical* range (about 26%). Thus, the ASD+ADHD Group obtained high percentages of dysfunction on all the SPM subscales: SOC (89.4%), TOT (73.7%), and Planning and Ideas (68.4%), including all the different sensory modalities subscales: BOD (84.3%), Vision (73.7%), Hearing (68.4%), Balance and motion (68.4%), and Touch (63.2%), according to the information provided by the parents of these children. Regarding the ADHD Group, about 50% of the participants obtained scores within the *Typical* range, and the other 50% obtained scores within the *Some Problems* and *Definite Dysfunction* ranges. The most affected sensory systems for the ADHD Group were BOD (52.6%), Balance and motion (52.6%), and Vision (52.6%). As for the ASD Group, 53.58% of the participants obtained scores within the *Typical* range, and 46.42% obtained scores within the *Some Problems* and *Definite Dysfunction* ranges. The most affected sensory system for the ASD Group was Hearing (61.9%).

Comparing the three groups with neurodevelopmental disorders, in general terms, the most affected group was the ASD+ADHD Group, which obtained worse scores than the ASD Group on the BOD subscale, and worse scores than the ADHD Group on the SOC subscale. The ADHD Group obtained worse scores than the ASD Group on the BOD subscale, whereas the ASD Group did not obtain worse scores than the ADHD Group on any subscale.

### Group differences in the classroom context

Statistically significant differences among the four groups were found, as revealed by both the MANOVA performed with the scores obtained on the *SPM-Main Classroom Form* [Wilk's Lambda (λ) = 0.237; *F*_(7, 21)_ = 7.15; *p* < 0.001; η^2^_*p*_ = 0.381], and the ANOVA performed with the scores obtained on the TOT subscale [*F*_(3, 86)_ = 17.91; *p* < 0.001; η^2^_*p*_ = 0.385]. As Table 3 shows, the teachers of the children with neurodevelopmental disorders (ASD and/or ADHD) evaluated their pupils' characteristics of SP, SOC, and praxis as significantly more dysfunctional than the teachers of the children in the CG, except on the HEA subscale, where there were no statistically significant differences between the CG and the ADHD Group, and the BOD subscale, where there were no differences between the CG and the ASD Group.

**Table 3:** T-score means, standard deviations, and *F*-values for SPM-classroom form subscales.

		**ASD group**	**ADHD group**	**ASD+ADHD Group**	**Comparison group**	***F*_(3, 86)_**	**η^2^*_*p*_***	**Group differences**
VIS	*M*	62.00	60.34	63.10	51.81	11.10^**^	0.279	C<ADHD, ASD, ASD+ADHD
	*SD*	7.86	6.03	7.13	9.00			
HEA	*M*	60.57	54.29	65.05	47.78	19.42^**^	0.404	C<ASD, ASD+ADHD;
	*SD*	10.83	7.94	6.48	7.62			ADHD<ASD + ADHD
TOU	*M*	65.90	61.58	63.57	49.52	13.95^**^	0.327	C<ADHD, ASD+ADHD, ASD
	*SD*	6.92	14.66	8.33	7.73			
BOD	*M*	56.33	61.20	61.95	48.30	11.63^**^	0.289	C<ADHD, ASD+ADHD
	*SD*	7.70	13.01	8.60	6.54			
BAL	*M*	56.86	59.68	58.67	46.67	9.21^**^	0.243	C<ASD, ASD+ADHD, ADHD
	*SD*	8.40	13.76	9.09	7.64			
TOT	*M*	62.19	61.58	64.62	48.74	17.91^**^	0.385	C<ADHD, ASD, ASD+ADHD
	*SD*	6.90	12.14	6.56	7.30			
SOC	*M*	69.81	58.87	73.10	49.56	49.72^**^	0.634	C<ADHD, ASD, ASD+ADHD;
	*SD*	8.41	5.94	6.36	8.40			ADHD<ASD, ASD + ADHD
PLA	*M*	65.00	61.44	67.62	49.93	26.06^**^	0.476	C<ADHD, ASD, ASD+ADHD
	*SD*	5.86	7.49	7.91	8.58			

VIS, Vision; HEA, hearing; TOU, touch; BOD, body awareness; BAL, balance and motion; TOT, total sensory systems; SOC, social participation; PLA, planning and ideas. A+A = ASD+ADHD Group; C, comparison group.

** *p < 0.013 Holm-Bonferroni correction of critical p-values when performing multiple comparisons*.

In order to obtain sensory profiles of each group in this context, we analyzed the percentages of scores obtained by each group in each of the three SPM interpretative ranges, and we noted that about 87.5% of the CG obtained scores in the *Typical* range, whereas a small percentage (about 12.5%) obtained scores in the *Some Problems* and *Definite Dysfunction* range. By contrast, the ASD+ADHD Group obtained the highest scores (about 73%) in the *Some Problems* and *Definite Dysfunction* ranges, whereas small percentages were obtained in the *Typical* range (about 27%) Thus, the ASD+ADHD Group obtained high percentages of dysfunction on the main SPM subscales: SOC (94.7%), Planning and Ideas (84.2%), and TOT (79%), and the sensory modalities subscales on which the highest percentages of dysfunction were obtained were: Hearing (84.2%), Touch (73.7%), and Vision (73.7%), according to the information provided by the teachers of these children. Regarding the ADHD Group, about 48% of the participants obtained scores within the *Typical* range, and about 52% obtained scores within the *Some Problems* and *Definite Dysfunction* ranges. The ADHD Group obtained a high percentage of dysfunction on the Planning and Ideas subscale (68.4%), and the most affected sensory systems were Touch (52.6%) and Vision (52.6%). As for the ASD Group, about 38% of the participants obtained scores within the *Typical* range, and about 62% obtained scores within the *Some Problems* and *Definite Dysfunction* ranges. The ASD Group also obtained high percentages of dysfunction on the SOC (90.4%) and Planning and Ideas (85.7%) subscales, and the most affected sensory system was Touch (81%).

Comparing the three groups with neurodevelopmental disorders, the most affected groups were the ASD+ADHD and ASD groups because the ASD+ADHD Group did not obtain worse scores than the ASD Group on any subscale. The ASD+ADHD Group obtained worse scores than the ADHD Group on the HEA and SOC subscales, and the ASD Group obtained worse scores than the ADHD Group on the SOC subscale, but the ADHD Group did not obtain worse scores than the ASD Group on any subscale.

### Intra-group differences

To compare the parent report with the teacher report, a MANOVA for repeated measures was performed in each of the four groups. The only group in which the MANOVA revealed statistically significant differences between the two informants was the ASD Group [Wilk's Lambda (λ) = 0.254; *F*_(8, 13)_ = 4.77; *p* = 0.006; η^2^ = 0.746]. These differences were found on the Touch (*p* = 0.001), SOC (*p* < 0.001), and Planning and Ideas (*p* = 0.004) subscales, with the teachers reporting higher dysfunction than the parents in all three cases.

## Discussion

As expected, the three groups of children with neurodevelopmental disorders obtained higher levels of dysfunction than the group of children with typical development on most of the SPM subscales (including praxis and SOC) both in the home and classroom contexts, with some exceptions. Thus, in both contexts the CG did not obtain differences with regard to the ASD Group on the BOD subscale, nor with respect to the ADHD Group in the hearing subscale. In addition to this, in the family context, there were no differences between the CG and the ASD Group on the touch and balance and motion subscales. In all these cases, moreover, the dysfunction percentages obtained were low. However, the comorbid group (ASD+ADHD) did obtain differences with regard to the CG on all the SPM subscales in both contexts, confirming the hypothesis that these two groups (ASD+ADHD and typical development) present the most different sensory profiles.

Comparing the three groups with neurodevelopmental disorders, there were differences according to the context. On the one hand, in the home context, the comorbid group (ASD+ADHD) was clearly the most affected group, obtaining high percentages of dysfunction on all the SPM subscales. On the BOD subscale, the dysfunction was similar to that of the ADHD group, and in both groups it was higher than that of the ASD group. This result suggests that difficulties in proprioception –the ability to sense the position in space of limbs, fingers, and other parts of the body- may be a sensory characteristic inherent to ADHD symptomatology, coinciding with previous studies (Shum and Pang, 2009; Jung et al., 2014). Moreover, the internal modalities (BOD, balance and motion) were among the most affected in both the comorbid and ADHD groups, obtaining high percentages of dysfunction. In contrast, it has been suggested that the ASD condition could be associated with a greater reliance on proprioceptive information, so that individuals with ASD may preferentially pay attention to internal sensory cues (Baum et al., 2015). In fact, the ASD group obtained the highest percentages within the typical range for the internal modalities (BOD, balance and motion) in the home context.

As for the external sensory modalities, very high percentages of dysfunction were found in the two groups of ADHD for vision and in the two groups of ASD for hearing. However, in the family context there were no differences between the three groups with neurodevelopmental disorders, so we cannot associate the condition of ADHD with a visual processing dysfunction (as it had been suggested in some previous studies, e.g., Shum and Pang, 2009; Jung et al., 2014) nor the condition of ASD with an auditory processing dysfunction (as it had been also suggested in some previous studies, e.g., Tomchek and Dunn, 2007; Ashburner et al., 2008; Wiggins et al., 2009; Fernández-Andrés et al., 2015).

Regarding praxis, there were no differences between the three groups with neurodevelopmental disorders. Contrary to expectations, the comorbid group did not present more difficulties than the other two groups in motor planning and ideation, which is also not in accordance with the result obtained by Unterrainer et al. (2016), who found more planning problems in ASD than in ADHD and ASD+ADHD.

Regarding SOC, the comorbid group presented more dysfunction than the ADHD group, confirming the hypothesis that social functioning difficulties are exacerbated in the comorbid condition compared to the ADHD condition, being this result novel. However, it was not found more social functioning difficulties in children with ASD+ADHD than in those with ASD alone, what is not in favor of the hypothesized results, which previously had also obtained Rao and Landa (2014). The comorbid condition, therefore, does not seem to have an additive effect on social difficulties, regarding the condition of ASD, in the family context.

On the other hand, in the classroom context, the ASD+ADHD and ASD groups were the most affected groups because there was no subscale on which the ASD+ADHD Group obtained worse scores than the ASD group. Moreover, there was no subscale on which the ADHD group obtained worse scores than the comorbid and ASD groups. The ASD+ADHD and ASD groups obtained high percentages of dysfunction on all the SPM subscales. Regarding the sensory modalities, touch processing was highly impaired in all three groups with neurodevelopmental disorders, which reinforces the result obtained in previous studies revealing the high prevalence of dysfunctions in this sensory system in ASD (Tomchek and Dunn, 2007; Ashburner et al., 2008; Wiggins et al., 2009; Fernández-Andrés et al., 2015) and ADHD (Parush et al., 2007; Ghanizadeh, 2008). This result could be related to the fact that in the classroom children are usually exposed to unpredictable tactile input that may become invasive for them (Dunn et al., 2002), especially for children with these neurodevelopmental disorders. However, although the comorbid condition (ASD+ADHD) was also associated with touch processing difficulties, there was no additive effect on the difficulties in this sensory system with respect to each condition separately, at least in the classroom context.

Auditory processing was the sensory system where the comorbid group obtained the highest percentage of dysfunction, significantly higher than in the ADHD group. In contrast, the ASD group did not obtain differences in this sensory system with respect to the other two neurodevelopmental disorders groups'. Thus, the ADHD symptomatology added to the ASD condition could exacerbate the auditory processing difficulties that are common in children with some neurodevelopmental disorders, especially in the classroom context, where many of the explanations and activities include verbal information.

As for the higher functions, the comorbid and ASD groups obtained very high percentages of dysfunction, above 90% in the case of SOC and above 84% in the case of praxis. However, there were no differences in praxis compared to the ADHD group, probably because of the executive dysfunction attributed to both the ADHD (Barkley, 1998) and ASD (Ozonoff, 1997; Hill, 2004) conditions. Therefore, the comorbid condition did not have an additive effect on praxis difficulties, with respect to each condition separately. In the case of SOC, both groups of children with ASD showed more difficulties than the ADHD group. Thus, in this case, the comorbid condition had an additive effect on the social difficulties with respect to ADHD, but not with respect to ASD. Therefore, social difficulties attributed to the ASD condition, which in fact are one of its diagnostic criteria (American Psychiatric Association, 2013), are greater according to the children teachers' than their parents. Nonetheless, this result does not mean that social difficulties are greater in class. These difficulties are pervasive in both contexts, but it is possible that the family has spent many years adjusting to and becoming familiar with these social difficulties, which would explain the possible difference in the perceptions of parents and teachers.

In sum, in agreement with our hypothesis, the comorbid group was clearly the most affected in the home context. The parents' perception of their child's SP difficulties might be greater in the comorbid group because ADHD symptomatology is one of the aspects that causes more parental stress in parents of children with ASD (Pastor-Cerezuela et al., 2016). In the classroom context, however, considering the teachers' point of view, the manifestation of sensory and higher function difficulties is greater in the case of the two autism groups (comorbid and ASD), with the two groups being the most affected, and no significant differences between them.

Finally, regarding the comparison of the information from the parents and teachers in each group, the only group where differences were found was the ASD group. In this group, the teachers reported greater dysfunction than the parents, particularly on the Touch, Social Participation, and Planning and Ideas subscales, in line with previous research (Fernández-Andrés et al., 2015). However, contrary to our expectations, none of the two other groups with neurodevelopmental disorders obtained differences between the two contexts on any of the SPM subscales.

We hypothesize that a possible explanation for this result could be related to the hyper-selectivity (or the detail-focused style of processing) that people with ASD show, as proposed in the framework of the Weak Central Coherence Theory (Frith and Happé, 1994) and the Enhanced Perceptual Functioning Theory (Mottron and Burack, 2001). According to these theoretical approaches, hyper-selectivity, or the ability to keep the focus of attention on a particular aspect of some detail in the context (concentrated and narrow attention focus), is a unique and characteristic feature of ASD. In this case, contextual hyper-selectivity would be associated with a manifestation of certain behaviors –related to the sensory level and high functions- that would be substantially different depending on the context (family-school). This context hyper-selectivity –as a unique and characteristic feature of ASD- would be manifested in the case of the ASD Group, but not in the comorbid group, perhaps due to the comorbid symptoms of inattention and hyperactivity/impulsivity in the ASD+ADHD group.

### Study limitations

Our study has several limitations. First, children with serious behavioral problems or very low cognitive functioning were not part of the sample, so that the autism spectrum was not fully represented. Second, there is a lack of information about whether children had received or were receiving some kind of sensory intervention at the time of the evaluation. Third, the evaluation measures were reported measures, leading to possible biases. Fourth, although the SPM assesses higher processes and allows a direct comparison of performance in different developmental contexts, it does not differentiate between over-responsiveness, under-responsiveness and sensory seeking across modalities, so that it may be necessary to use other complementary instruments, such as the Sensory Profile, to plan an effective individual intervention. Fifth, groups differ by class size and also by class type, so it is possible that this aspect impact the teacher ratings of children's behavior. Finally, this research used cross-sectional data and did not study the variables over time.

## Conclusion, practical implications, and future research prospects

Children with ASD and/or ADHD can present SP impairments in different contexts, which may contribute to inappropriate behavioral and learning responses. According to the results obtained in this work, specific intervention programs should be developed to improve the sensory challenges faced by children with different diagnoses. Thus, intervention programs for children with ASD should include activities to enhance auditory and tactile processing problems, whereas intervention programs for children with ADHD should enhance proprioception, tactile, and visual processing difficulties. In both cases, intervention should also take into account the high function problems these children experience. In the case of a comorbid diagnosis, it would be advisable to implement strategies to improve BOD and balance and motion difficulties, as well as tactile, auditory, and visual difficulties. Despite these preliminary results, it must be taken into account that sensory interventions have to be individualized treatments, and further research is needed to determine a differential sensory pattern for children with ASD, ADHD, and a comorbid ASD+ADHD diagnosis.

Earlier detection and management of SP problems are essential because research shows that children and adolescents with neurodevelopmental disorders have responded positively to sensory integration therapy (Schaaf et al., 2013; Tomchek et al., 2017). This is a child-centered intervention that uses playful and goal-directed activities that provide a “just-right” sensory motor challenge, scaffolding the child's emerging skill (Case-Smith et al., 2014). This approach enhances intrinsic motivation, and it is especially effective in reducing self-stimulating behaviors and aggression (Smith et al., 2005). Apart from sensory integration therapy, it is also important for children to learn relaxation and insight techniques in order to start feeling their bodies and be able to respond to stimuli more consciously. These techniques can help to create a space or response delay between thoughts and actions, which may, in turn, reduce the number of disruptive behaviors that some of these children present.

It is also essential to evaluate different contexts, such as the home and the school, as each context contains unique characteristics that can support and/or create challenges for the child's performance (Dunn et al., 2002). Likewise, it is necessary for occupational therapists to work cooperatively with parents and teachers, not only to identify the children's SP impairments, but also to help them understand how these children experience the world and teach them some strategies. Most of the published studies on sensory problems rely on parent and/or teacher reports. Although, as previously discussed, some studies have used objective measures, such as observational and performance tasks, further investigation is required in order to improve the differentiation between SP problems and other disorders or problems, and shed light on the relationship between SP and cognitive functioning in neurodevelopmental disorders. More longitudinal studies are needed, as suggested in McCormick et al. (2016), in order to test the SP development of children with age. In consonance with the Marco et al. (2011) study, more research about neurophysiological profiles of SP in ASD and ADHD would also serve as valuable biomarkers for diagnosis and for monitoring therapeutic interventions.

## Author contributions

Conceived and designed the work: PS, MF, GP. Acquired data: FG, RT. Coded data: PS, MF, RT. Corrected data: MF, FG, RT. Analyzed data: MF, GP. Interpreted data: PS, GP, FG. Wrote the paper: PS, GP, MF. Drafted the article and revised it critically: GP, FG, RT.

### Conflict of interest statement

The authors declare that the research was conducted in the absence of any commercial or financial relationships that could be construed as a potential conflict of interest.
