# Corrigendum: Sensory Processing in Children with Autism Spectrum Disorder and/or Attention Deficit Hyperactivity Disorder in the Home and Classroom Contexts

**DOI:** 10.3389/fpsyg.2019.00443

**Published:** 2019-03-05

**Authors:** Pilar Sanz-Cervera, Gemma Pastor-Cerezuela, Francisco González-Sala, Raúl Tárraga-Mínguez, Maria-Inmaculada Fernández-Andrés

**Affiliations:** ^1^Teaching and Scholastic Organization Department, Faculty of Philosophy and Educational Sciences, University of Valencia, Valencia, Spain; ^2^Basic Psychology Department, Faculty of Psychology, University of Valencia, Valencia, Spain; ^3^Developmental and Educational Psychology Department, Faculty of Psychology, University of Valencia, Valencia, Spain

**Keywords:** Attention Deficit/Hyperactivity Disorder (ADHD), Autism Spectrum Disorder (ASD), higher functions, home and classroom contexts, sensory processing, Sensory Processing Measure (SPM)

There is an error in the Funding statement. The correct number for the Ministry of Economy, Industry and Competitiveness is “Grant EDU-2016-78867R AEI/FEDER, EU.”

Additionally, in the original article we neglected to mention the city and country the research was conducted in.

A correction has been made to the Materials and Methods, Comparison Group, Paragraph two:

“All of the children attended the same schools. Children from the ASD Group and the ASD+ADHD Group were attending schools with specific classrooms where the Treatment and Education of Autistic and Related Communication Handicapped Children (TEACCH) methodology was used. These are special classrooms integrated in regular state schools in Valencia (Spain), where students with disorders affecting language and communication are enrolled. In these classrooms there are a maximum of 8 children attended by three specialists: a special education teacher, a hearing and language teacher and an educator. These children are not all the time in these special classrooms, but they share their timetable both in these classrooms and in their corresponding mainstream classroom, where they are usually accompanied by one of the specialists who work in the special classrooms. Children from the ADHD Group and the CG, however, were attending the same schools as the children in the ASD and ASD+ADHD Groups, but in the regular modality.”

The authors apologize for these errors and state that this does not change the scientific conclusions of the article in any way. The original article has been updated.

